# Water Quality Degradation Due to Heavy Metal Contamination: Health Impacts and Eco-Friendly Approaches for Heavy Metal Remediation

**DOI:** 10.3390/toxics11100828

**Published:** 2023-09-30

**Authors:** Peng Zhang, Mingjie Yang, Jingjing Lan, Yan Huang, Jinxi Zhang, Shuangshuang Huang, Yashi Yang, Junjie Ru

**Affiliations:** 1 School of Hydraulic Engineering, Wanjiang University of Technology, Ma’anshan 243031, China; wjymj2018@gmail.com (M.Y.); ljjwanjianggong@gmail.com (J.L.); yan.huang0113@gmail.com (Y.H.); zhang.jingxi001@gmail.com (J.Z.); hang.ss1991@gmail.com (S.H.); yang.yashi1314@gmail.com (Y.Y.); 2 College of Hydrology and Water Resources, Hohai University, Nanjing 210098, China

**Keywords:** water contamination, heavy metals, health impacts of heavy metals, heavy metals removal

## Abstract

Water quality depends on its physicochemical and biological parameters. Changes in parameters such as pH, temperature, and essential and non-essential trace metals in water can render it unfit for human use. Moreover, the characteristics of the local environment, geological processes, geochemistry, and hydrological properties of water sources also affect water quality. Generally, groundwater is utilized for drinking purposes all over the globe. The surface is also utilized for human use and industrial purposes. There are several natural and anthropogenic activities responsible for the heavy metal contamination of water. Industrial sources, including coal washery, steel industry, food processing industry, plastic processing, metallic work, leather tanning, etc., are responsible for heavy metal contamination in water. Domestic and agricultural waste is also responsible for hazardous metallic contamination in water. Contaminated water with heavy metal ions like Cr (VI), Cd (II), Pb (II), As (V and III), Hg (II), Ni (II), and Cu (II) is responsible for several health issues in humans, like liver failure, kidney damage, gastric and skin cancer, mental disorders and harmful effects on the reproductive system. Hence, the evaluation of heavy metal contamination in water and its removal is needed. There are several physicochemical methods that are available for the removal of heavy metals from water, but these methods are expensive and generate large amounts of secondary pollutants. Biological methods are considered cost-effective and eco-friendly methods for the remediation of metallic contaminants from water. In this review, we focused on water contamination with toxic heavy metals and their toxicity and eco-friendly bioremediation approaches.

## 1. Introduction

Environmental contamination refers to the presence of unwanted materials in the air, water, and soil that exceed the permissible limit. Furthermore, it is characterized as an undesirable alteration in the natural environment that results in harmful consequences for both flora and fauna [1]. Environmental pollutants are categorized into several classes, such as organic and inorganic pollutants and microbial contamination [2]. Organic pollutants can be defined as naturally occurring biodegradable substances and anthropogenic activities. A few examples of these pollutants are food and agro-waste, polycyclic aromatic hydrocarbons (PAHs), domestic and dairy waste products, and polychlorinated biphenyls (PCBs) [3]. Biological pollution is generally caused by bacteria, viruses, molds, mites, cockroaches, pollen, and fungi [4]. The pollution caused by human beings impacts terrestrial and aquatic ecosystems [5]. Leather tanning, chrome plating, battery industries, pigment industries, and chemicals (fertilizers, pesticides, and insecticides) used in the agriculture field add pollutants to the environment. Inorganic contaminations such as heavy metal ions originate from natural or anthropogenic activities [6,7,8].

Water degradation can occur due to dissolved toxic heavy metals in the water [9]. This causes water to become unsuitable for human use [10]. Bisimwa et al. [11] have observed that the quality of surface water sources is significantly affected by wastewater effluents. Meanwhile, the dominant source of heavy metals in surface water sources appears to be contaminated by several industrial processes [12]. Changes in water quality in surface waters are also influenced by natural biogeochemical processes and hydrological systems in river ecosystems [13,14]. Heavy metal contamination in water sources, particularly groundwater and surface water, has several harmful impacts on the human community. The proliferation of urban landscapes, industrial development, and chemical fertilizer use in agriculture has resulted in an upsurge in toxic metallic contaminants in aquatic ecosystems via industrial wastewater, urban drainage networks, and stormwater runoff management systems [15]. The heavy metal contamination present in the water decreases the quality of water. There are several techniques used to determine heavy metal contamination in water, such as inductively coupled plasma mass spectrometry (ICP-MS) and atomic absorption spectroscopic (AAS) [16,17]. The determination of heavy metal contamination in water is helpful in the explanation of water quality. The presence of heavy metals, such as Cr (VI), Cd (II), Pb (II), As (V and III), Hg (II), Ni (II), Cu (II), etc., in wastewater poses a significant threat as these water pollutants tend to accumulate in the food chain and drinking water sources [18]. Heavy metal contamination causes several adverse effects, like kidney damage, liver failure, gastric problem, mental retardation, and reproductive effects in humans. Heavy metals also result in carcinogenic effects in humans [19,20]. 

Several methodologies are accessible for the removal of heavy metals from water. Physicochemical methods, such as reverse osmosis, chemical precipitation, membrane filtration, etc., are frequently used for heavy metal removal [21]. These methods are cost-effective and generate large amounts of secondary pollutants [22]. Moreover, these methods are only effective relative to higher concentrations of heavy metal (above 2 mM) ions in the water [23,24]. Considering the disadvantages of physical and chemical water treatment methodologies, there is an urgent need for suitable eco-friendly and cost-effective alternatives to these physicochemical methods for the removal of heavy metals from water [25]. 

Biological methods, such as biosorption, bioaccumulation, bioreduction, phytoremediation, and mycoremediation, are effective alternatives to physicochemical methods. These biological methods also work on low-cost and eco-friendly methodologies [26]. These methods can be divided into two categories: metabolically independent and metabolically dependent methods. Biosorption is a metabolically independent method based on dead biomass and agriculture residue [27]. A variety of biomass, such as rice husk, wheat husk, banana peels, and microbial biomass, are used as biosorbents [28]. Metabolically dependent heavy metal remediation is based on living microorganisms, and several metabolic reactions are involved in this process. Living microbes, like bacteria and fungi, are considered effective remediating agents [29]. The heavy metals present in the aqueous medium bind to the microbial surface and enter the cell through the cell surface receptor [30]. Antioxidants, such as superoxide dismutase (SOD), catalase (CAT), and glutathione S-transferases (GST), are present in the bacterial cell and are neutralized relative to the heavy-metal-mediated oxidative stress in the cell. These antioxidants also help in heavy metal bioremediation by minimizing heavy metal stress in microbial cells [30].

This review is focused on water contamination with heavy metals, major sources of heavy metals, and toxicity. This review is also focused on heavy metal remediation technologies. 

## 2. Water Quality Assessment

Water quality criteria are established with the utmost consideration of variables that are known to characterize the quality of water, as indicated by [31]. Several water quality criteria are put in place to ensure that the maximum concentration level of a particular substance in a specific medium, whether it is water, sediment, or biota, does not pose any harm to the users of the said medium, particularly when the medium is used continuously for a specific purpose. For some water quality variables, including dissolved oxygen (DO), the criteria are based on the minimum acceptable concentration that is required to maintain biological functions [31]. The quality of water is significantly influenced by physicochemical parameters, which include but are not limited to pH, temperature, conductivity, biochemical oxygen demand (BOD), chemical oxygen demand (COD), total dissolved solids (TDS), minerals, and heavy metal concentration. The World Health Organization recommends the maximum allowable limits for water physicochemical parameters, as shown in Table 1.

Criteria are commonly established for raw water with respect to its utilization as a water source for drinking water supplies, agriculture, and recreation or its utilization in dwellings for biological communities. Moreover, criteria may also be formulated for the operational efficiency of aquatic ecosystems at large [33]. The preservation of these water utilities typically necessitates distinct requisites for water quality; thus, the corresponding water quality criteria usually vary for each use. According to WHO, the pH range of drinking water should be 6–9, and the temperature of drinking water should be suitable at 25 °C [33,34]. 

Following the exhaustive assessment of the ambient water quality for particular water quality objectives, it is inevitable that any inadequacies shall mandate the implementation of appropriate actions to effectively manage contaminants for discharges, including discharges in upstream sections [35]. It is noteworthy that this particular mechanism also serves to facilitate the development and establishment of industries, thus underscoring its vital importance in the overall scheme of environmental management. It should be explicitly stated that under no circumstances are industries allowed to discharge any kind of effluent into water bodies [35,36].

The evaluation of water quality has emerged as a pivotal aspect of water management in recent times due to the significant global importance attached to escalating concerns and the awareness of environmental and health-related impacts over the years [37]. With the current shift towards decentralization, numerous agencies have come into existence to assess water quality, including central boards, state public health engineering departments, state boards, and several governmental agencies that operate externally [38]. The measurement of water quality constitutes a vital and preliminary phase of water management and contamination control. Contamination in water is increasing beyond the permissible limit relative to water components, including nitrate, phosphate, magnesium, and heavy metals present in the water [35,36,37]. The physicochemical parameters of water can be characterized using different methods. There are several water quality parameters in drinking water, and their detection analytical methods are shown in Table 2.

## 3. Heavy Metal Contamination and Water Quality Degradation

There are two types of heavy metal contamination in water, namely point sources and non-point sources. Surface water sources, including rivers, lakes, and ponds, can be polluted by effluent discharged from a point source via overflow or drainage. In non-point sources, heavy metal contaminants are carried into surface water sources by rainwater runoff (surface runoff) [43]. The heavy metal contamination load (CL) in water can be calculated using Equation (1):

(1) CL=HC×Q×86.4

where CL is heavy metal contamination load (kg/day), HC is heavy metal contamination in contaminated water (mg/L), and Q is the flow rate (m^3^/s). 

Insufficient water supplies and water treatment facilities, industrialization, agricultural activities, and natural factors are major causes of heavy metal contamination in water. Heavy metal contamination in water is caused by industries such as distilleries, tanneries, pulp and paper industries, textile industries, food industries, iron and steel industries, nuclear industries, etc. [44,45,46,47]. The second reason is that agriculture is closely associated with water contamination. Agricultural-mediated water contamination is a result of pesticides, fertilizers, and herbicides used in agriculture [48]. 

The existence of heavy metal contamination in water engenders potential hazards to the ecosystem and well-being in the water-deprived areas of emerging economies, such as China and India. In regions with untreated or partially treated water, the water is extensively employed for agricultural irrigation purposes [49]. As a result of the imbalance, some areas in developing countries have used wastewater irrigation for years to meet agricultural production water demands, leading to serious land and food contamination with heavy metals and posing serious health and food safety risks [50,51]. The consumption of potable water is known for heavy metals that have been found to impose a deleterious effect on human health. A wastewater-irrigated village in India has a higher morbidity rate than a village that uses normal water. Natural factors are also involved in heavy metal water contamination. Trace elements may be attributed to natural weathering or manufacturing processes [52]. A wide range of heavy metal sources can be found in surface water, including both point and non-point sources [53]. 

### 3.1. Anthropogenic Source of Heavy Metal Contamination

The anthropogenic sources of water contamination are defined as heavy metal contamination in water that is caused by human activity. Several industrial, agricultural, and domestic activities are responsible for heavy metal contamination in water [54]. Urban centers situated along or close to the river surface water sources serve as a prime illustration of water contamination, wherein industrial or domestic effluents containing toxic heavy metals are discharged directly into the river, thereby unwittingly contaminating it with hazardous substances. Anthropogenic sources of water contamination fall into several categories, some of which are described below. 

#### 3.1.1. Industrial Sources

There has been an alarming increase in heavy metal contamination in the surface and groundwater as a result of industrial waste over the past decade [55]. According to a survey conducted by authorities [56], a report by the Central Pollution Control Board (CPCB) in India has indicated that a daily industrial wastewater equivalent of 260 million liters is released into the Ganga River. Industrial effluents release a variety of toxic chemicals, organic and inorganic substances, toxic solvents, and volatile organic chemicals. Heavy metal contamination in water occurs if these industrial effluents are not completely treated before entering aquatic ecosystems [45]. The industrial sector contributes significantly to the presence of harmful heavy metals, such as As (III), Cd (II), Pb (II), Cr (VI), Ni (II), Hg (II), and Cu (II) in water [46]. Industrial wastewater production has gradually increased with the acceleration of urbanization [47]. There are industries, such as tanning, chrome plating, coal industries, metalworking, plastic processing, food industries, ceramic application, and agrochemical waste industries, that are responsible for the addition of the number of hazardous heavy metals in water [57]. The discharge of industrial effluents containing heavy metals into water bodies, whether partially treated or untreated, has been identified as a major contributor to various health concerns experienced by humans and other animals [58]. Coal, classified as a fossil fuel, possesses a preeminent degree of importance within the energy sector and is commonly acknowledged as a predominant energy resource in various nations, including but not limited to India, China, Nepal, Pakistan, and several others [59]. In contrast to alternative sources of energy, coal is easily accessible and comparatively affordable, rendering it the favored option for a multitude of sectors [59]. Nevertheless, the treatment of coal leads to a considerable quantity of hazardous materials being released as coal washery effluents (CWEs), incorporating heavy metal ions like As, Cd, Cr, and Pb. This emission results in the widespread contamination of the environment [60].

#### 3.1.2. Domestic Sources

How much domestic waste impacts heavy metal contamination is dependent on the efficiency of wastewater collection systems. Furthermore, waste transportation systems differ in type and length [61,62]. The main components of domestic waste are organic matter and microorganisms. Aside from these ingredients, domestic sources also contribute salts, chlorides, nutrients, detergents, oil, grease, and heavy metals. The Yamuna River in India is heavily contaminated with domestic waste. Large urban centers that dump domestic waste into rivers are mainly responsible for domestic contamination [63].

#### 3.1.3. Agricultural Sources of Pollution

When pollutants run off into a waterway, they are considered nonpoint sources [64]. Runoff from a field can carry fertilizers and pesticides containing several heavy metals into a stream [64,65]. The heavy metal contamination in the water due to agriculture is attributed to several key factors, namely agricultural residues, herbicides, fertilizers and pesticides, and excessive salts that result from the application of irrigation water [66]. The agricultural waste produced in the basin of the river and near other water bodies, like lakes and ponds, is naturally decomposed, eventually leaching toxic metals into the water [67,68]. Moreover, the presence of heavy metal contamination in surface water can also be attributed to the utilization of agricultural applications. There are several heavy metals, such as Cd (II), Pb (II), Cr (VI), As (III), Hg (II), Ni (II), Cu (II), and Zn (II), that enter the water cycle due to fertilizers, nutrients, and pesticide applications. Cd (II), Pb (II), Cr (VI), As (III), and Hg (II) are toxic to human and animal health [69].

#### 3.1.4. Dumping of Waste and Landfills

The matter of dumping and unsanitary waste landfills is an urgent concern that warrants scholarly attention. Municipal solid waste (MSW) encompasses household, healthcare, and industrial refuse; however, categorization is lacking as they are indiscriminately deposited into a single landfill [70]. The principal location for the disposal of solid waste is the landfill, where the consequences of environmental contamination and the proliferation of disease have been severe [71,72]. The transportation of leachate in open dumping sites is a predominant source of heavy metals in surface and groundwater, soil, and vegetation [73]. If flora absorbs heavy metals from polluted soil, the heavy metals are likely to be transferred to the human food chain via the ingestion of these plants [74]. Heavy metals like Cd (II), Cr (VI), Cu (II), Pb (II), Ni (II), and Zn (II) that are present in the food chain cause several health problems in living organisms [18,75]. 

### 3.2. Natural Source of Heavy Metal Contamination

There exists a multitude of naturally occurring origins that contribute to the contamination of heavy metals in the water. The principal natural sources of this toxic element are found within leaching, weathering, volcanic eruptions, etc. [76]. During the weathering process, rock, soil, and minerals are broken down by interactions between elements, such as oxygen, and water; living organisms; and the atmosphere. It is important to note that weathering is a process that takes place on site and does not involve the transport of weathered materials. It is also important to note that weathering can occur in many different ways, such as physically, biologically, and chemically [77]. As a result of physical weathering, heavy metals containing minerals are decomposed by high temperatures, water, ice, pressure, and other weathering microclimate conditions, which cause the metals to break down. By contrast, chemical weathering occurs when chemical elements in rocks react with microclimatic conditions, resulting in the release of heavy metals from the host rock to the atmosphere as a result of the chemical reactions described above [78]. As part of the biological process, rocks and soil molecules are broken down in the search for nutrients to support plant growth or microorganism growth in the soil [77,78]. Heavy metals are released from soil matrixes via leaching and the use of different leaching agents. There are multiple types of lechates, including water, acids, bases, and organic compounds [79]. Usually, rainfall and rock fractures on the surface of the ground can result in contact between the leachable fraction of the geological structure and aqueous media, which in turn leads to the leaching of metals from the geologic structure. In addition to pH, buffering minerals, solubility, and the susceptibility of rocks, there are also some factors to consider. Several processes affect leaching, including desorption, complexation, pH, redox, dissolved organic matter, and microbiological action [80].

## 4. Toxicity of Heavy Metals and Human Health

The degradation of both surface water sources and groundwater quality caused by industrial wastes, urban sewage, and agricultural runoff is a matter of concern. The determination of the suitability of groundwater for specific uses, such as irrigation, public water supply, industrial applications, and power generation, is highly dependent on groundwater [81]. The quality of groundwater is influenced by a variety of processes and reactions that occur, such as condensation in the atmosphere and the moment water is discharged by a well [82]. As a result, groundwater quality varies from one location to another with respect to the depth of the water table and from season to season. The amount and composition of dissolved solids, including heavy metals present in groundwater, primarily govern water quality [83]. The quality of groundwater is slowly but steadily declining worldwide. Hydrological, physical, chemical, and biological factors all play a significant role in determining groundwater quality [83]. 

Phytochemical and biochemical functions of plants, animals, and humans are affected by some trace metals, while other trace metals are toxic even in minute amounts [84]. Based on their health relevance, trace metals are classified as essential (Fe (II), Mn (II), Zn (II), and Cu (II)) and non-essential (Pb (II), Cd (II), and As (III)) [85]. Inhalation, ingestion, and dermal absorption are the three main pathways through which high concentrations of trace metals interact with humans. Human exposure to contaminated water occurs through the ingestion of drinking water and food, as well as dermal contact [86,87]. 

**Figure 1:**
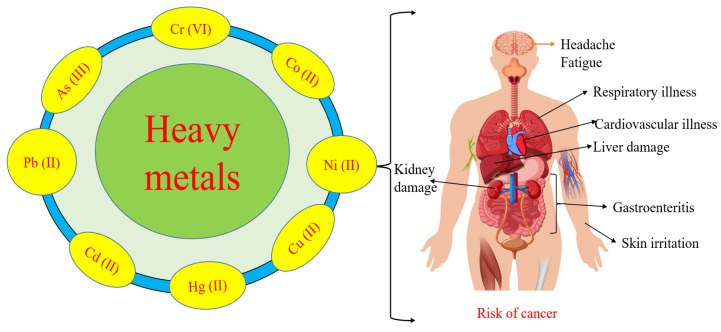
Water pollution and its harmful impacts on human health.

When heavy metal ions enter the human body through food or water, they initiate various processes in the body. Heavy metals such as Cr (VI), Pb (II), and As (III) interfere with metabolic pathways or inhibit enzymatic activities [88]. Cr (VI) ions can easily cross the cell membrane and are reduced in intracellular space via their lower oxidative stage. The reduction process generates oxidative stress in the cell, which is responsible for damage to proteins, DNA, and RNA [17]. Biological systems produce reactive oxygen species (ROS) when heavy metal ions bond with sulfhydryl groups to form reactive oxygen species. This oxidative stress causes macromolecules to be inactivated, which results in oxidative stress, and the glutathione levels in the body decrease [89]. Consequently, both humans and animals suffer from a wide range of harmful effects as a result of these pollutants. Congenital disorders, immune system problems, and cancer are among the problems that can result [90]. The health risks associated with heavy metal toxicity are summarized in Figure 1. 

Researchers have focused attention toward comprehending the impact of metallic pollutants, primarily focusing on their elevated toxicological behavior [91]. Although present in trace quantities in various ecosystems, these substances have been found to exert detrimental effects on ecological health. It is noteworthy that certain metal ions exhibit toxicity even at relatively lower concentrations, warranting further investigation into the understanding of their ecological ramifications [92]. Metals Cr (VI), As (III), Pb (III), Fe (II), Cd (II), Ni (II), Hg (II), and Co (II) are known to be highly toxic even in small amounts. However, some heavy metals, like Cr (II), Co (II), Zn (II), Fe (II), Mo (VI), K (I), and Cu (II), are essential and participate in various metabolic activities in living organisms [93]. However, the excess intake of these metals can cause harmful impacts on animals. The excess amount of heavy metal ions is not efficiently metabolized and instead accumulates in the intra- or extracellular regions of the body’s organs [92]. We have summarized some well-known heavy metals and their harmful health impacts in Table 3. 

The heavy metal ions mentioned in Table 3 show several health impacts. Pb (II), Cr (VI), Cd (II), As (III), and Ni (II) are carcinogenic and cause several types of cancers, including skin and gastric cancer. Moreover, Ni (II), Cd (II), and Hg (II) toxicity are associated with respiratory disorders. 

## 5. Removal of Heavy Metal Ions

There are several heavy metal removal methods used for the minimization of heavy metal contamination in water. Physicochemical methods like ion exchange, coagulation, precipitation, adsorption, membrane separation, and reverse osmosis are widely used for heavy metal removal [100]. The physicochemical methods are costly and generate secondary pollutants after the treatment of heavy metals from contaminated water. Another disadvantage of physicochemical methods is as follows: They are only effective when heavy metal concentrations are high in water (above 2 mM) [101,102]. Considering the disadvantages of these physicochemical methods, researchers are focused on the development of cost-effective and eco-friendly methods for removing heavy metals from contaminated water [103]. The advantages and limitations of some important heavy metal removal methods are discussed in Table 4.

Biological methods are suitable for heavy metal removal compared to other physicochemical methods. There is an urgent need to improve the efficiency of biological methods for heavy metals. The biological removal of heavy metals is very attractive to researchers due to its eco-friendly nature and cost-effective process. These methods are also effective at lower concentrations of heavy metals in the water [114]. Living organisms and materials obtained from the living world are utilized for the removal of heavy metal ions. Agriculture residue, plant-based biomass, green synthesized nanomaterial, and microorganisms have potential applications in the removal of heavy metals from water [115,116]. Living organisms, including fungi, algae, and bacteria, have emerging applications due to their unique properties. There are several biological methods, such as biosorption, bacterial bioremediation, and mycoremediation. Phytoremediation is characterized into two categories, metabolically dependent and metabolically independent, based on dead or live biomass used for heavy metal removal [117].

### 5.1. Metabolically Independent Methods for Heavy Metal Removal

**Figure 2:**
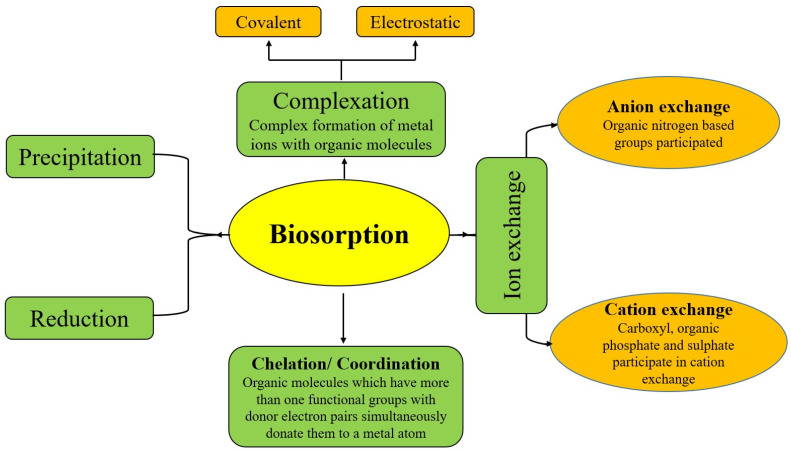
Mechanism of heavy metal biosorption.

Biosorption is generally carried out by dead biomass, and this phenomenon is known as a metabolically independent process. Plant biomass, agriculture residue, green synthesized nanomaterial, dead microbial biomass, and algal biomass are used for the removal of heavy metals in the biosorption process. The material used for the removal of heavy metal ions from water is known as a biosorbent [118]. The processes involved in biosorption and their mechanisms are summarized in Figure 2.

As heavy metal ions and protons exchange ions at binding sites during biosorption, ion exchange occurs. Ion exchange is mediated by anion and cation exchange mechanisms [119]. Covalent binding and electrostatic forces are involved in the coordination and formation of complexes. Heavy metals interact with active functional surface groups on the surface of the biosorbent to form complexes [120]. When pH changes or heavy metal ion concentrations increase in a solution, precipitation occurs. Biomass can produce compounds that play a key role in the precipitation of heavy metal ions [121]. The formation of chelates, which involves the complex formation of heavy metal ions and the surface ligands of biosorbents, is another important phenomenon known as chelation. The mechanism of biosorption also involves the reduction of heavy metal ions, with the more harmful metal species being reduced into less toxic forms, such as the reduction of Cr (VI) into Cr (III) [122].

The pKa value of the medium is also considered an important parameter for heavy metal biosorption. The binding tendency of surface functional groups present on the biosorbent surface is affected by the pKa value of the solution [123]. Temperature, initial metal ion concentration, biosorbent dosage, and incubation times are the major components that affect to biosorption process. The selection of suitable biomass for the preparation of biosorption is a very important and memorable stage for the biosorption of heavy metals. Raw materials should be easily and widely available, cost-effective, and non-toxic [124]. The number and disparity of the functional groups present on the biosorbent’s surface are also considered for suitability relative to biosorption [125]. An important characteristic of an adsorbent is its surface morphology, which plays an important role in the adsorption of heavy metals. Compared to smooth surfaces, rough and porous surfaces provide a larger surface area on the biosorbent’s surface, which has proven beneficial for binding heavy metal ions to it [126]. The surface morphology of biosorbents along with their functional groups needs to be characterized to understand their functions. As a result, a wide range of characterization methods has been developed for the study of biosorbents, including Fourier transform infrared (FTIR), scanning electron microscopy (SEM), energy dispersive X-ray analysis (EDX), nuclear magnetic resonance (NMR), and X-ray diffraction (XRD) [127,128].

The morphology of the biosorbent also affects biosorption capacities. A rough and porous surface provides more space for the accommodation of heavy metals on the biosorbent’s surface [129]. The pH of the medium is an important factor to consider in the biosorption process. The competition between cationic heavy metal ions is commonly caused by a decrease in pH. The deprotonating of adsorbent surfaces occurs at elevated pH values and exposes the functional groups of the surfaces [129,130]. Desorption can also be used to regenerate biosorbents. Changing the pH of the medium allows metal ions to be recovered [131]. 

There are several biomass types that can be used as biosorbents for the biosorption of heavy metals. We have summarized a few important biosorbents and their heavy metal biosorption capacity in Table 5. 

A pine-cone-derived biosorbent has a higher affinity for Pb (II). It shows higher adsorption capacity (100.01 mg/g) compared to Cd (II) (78.73 mg/g), Cu (II) (33.55 mg/g), and Cr (VI) (57.36 mg/g). The surface modification of the biosorbent enhances adsorption capacities. The enhancement in the adsorption capacity might be because of the increased surface area of the modified adsorbent or a change in surface morphology. The adsorption capacity of the material can be increased by treating it with ethylenediaminetetraacetic acid (EDTA), sulfur, and triethylenetetramine [141,142]. A list of a few modified adsorbents and their heavy metal biosorption capacity is shown in Table 6.

### 5.2. Metabolically Dependent Approaches for Heavy Metal Removal

Metabolically dependent heavy metal removal is carried out by living organisms or plants. Bioaccumulation is a process in which heavy metal uptake is carried out by living microbes, and the metals are taken into their intracellular space [149,150]. The metabolically dependent heavy metal removal process is complex compared to the metabolically independent process because there are several mechanisms and metabolic reactions involved in this process [149]. The heavy metal bioaccumulation process is generally carried out by living organisms in water or soil [151]. The bioaccumulation process minimizes the other several processes involved in biosorption, such as collection, drying, and the preparation of adsorbents [152]. There are a few disadvantages with respect to living biomass-based bioremediation, and they include maintaining the culture and requirement of growth media for the microbial culture. Growth conditions, such as temperature and pH, are also required for living microbial cells. Therefore, growth conditions based on living microbial cells should be considered during bioremediation [153]. Another problem with microbial-based heavy metal removal is the competition between metal–metal and other pollutants present in the water. The pollutants present in the water can bind to the microbial surface and cause disturbances in heavy metal accumulation due to microbes [154]. Various bacteria, fungi, and algae have shown emerging applications for the removal of heavy metals from contaminated water [155,156]. We have summarized some potential microorganisms and their potential heavy metal removal efficiency in Table 7. 

**Figure 3:**
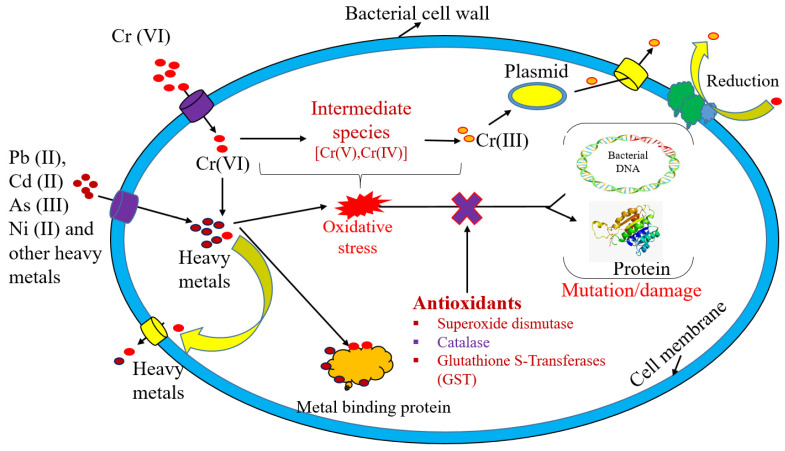
Bioremediation of Cr (VI) metal ions via bacteria and the remediation mechanism.

Among all living systems, microorganisms have excellent heavy metal tolerance and removal properties. Microorganisms such as bacteria can intake heavy metals through cell surface receptors and accumulate them in the cell [162]. *Pseudomonas alcaliphila* strain NEWG-2 was reported by El-Naggar et al. [163] to be 96.60% efficient in removing Cr (VI). Tekerlekopoulou et al. [164] used a mixed bacterial culture, including *Raoultella*, *Citrobacter*, *Klebsiella*, *Salmonella*, *Achromobacter*, and *Kerstersia* species, to achieve a high Cr(VI) removal rate (2 mg/L h) at 12.85 mg/L Cr(VI). Humphries et al. [165] observed that bacterial strains *Desulfovibrio vulgaris* NCIMB 8303 and *Microbacterium* sp. NCIMB 13,776 have 95% and 60% Cr (VI) removal efficiency relative to contaminated water. Ibrahim et al. [166] investigated heavy metal Cr(VI) reduction by extreme alkaliphilic *Amphibacillus* sp. KSUCr3, and a 62% reduction in efficiency was observed [166]. The bacterial species generally become resistant to heavy metal ions isolated from the contaminated sites with respect to the same heavy metals. Singh et al. [167] isolated a Cr (VI)-resistant bacterial stain named *Microbacterium paraoxydans* strain VSVM IIT(BHU). Singh et al. [167] isolated this bacterial strain from contaminated sites using coal washery effluents. Henson et al. [168] extracted a Cr (VI)-resistant bacterial species named *Microbacterium* sp. (Cr-K29). According to Henson et al. [168], this bacterial strain was able to reduce 88% Cr (VI) from the contaminated water. Bacteria follow a complex mechanism for the remediation of heavy metals. There are several functional groups on the bacterial surface involved in the binding of heavy metals on bacterial cell surfaces. Cell surface receptors on the bacteria cell participate in the binding and entry of heavy metal ions into bacterial cells [169]. The bioremediation of heavy metal ions by bacterial cells is shown in Figure 3.

When bacteria are exposed to toxic heavy metals, they produce ROS as a result of the release of reactive oxygen molecules. It has been well established in the literature that the detrimental effects of ROS on cell organelles and metabolic processes are well documented [170,171]. Thus, the antioxidant system, which comprises enzymes such as superoxide dismutase (SOD) and catalase within microorganisms, plays a key role in mitigating the damage caused by ROS, which is released as a consequence of ROS production in the cell [172]. In addition, several species of bacteria that can resist the toxic effects of heavy metals have been identified.

## 6. Technology Challenges and Future Perspectives

The biological methods of heavy metal bioremediation are cost-effective and eco-friendly, but these methods are time-consuming and laborious. In addition, the main challenge is the application of bioremediation methods at the commercial level for the treatment of water. More research is required in the area [173]. A variety of methods have been developed to enhance the removal of heavy metals from water, including the use of chelating agents and surfactants, the immobilization of bacteria on solid surfaces, and the use of composite materials like nanomaterials and biopolymers [174]. A biological remediation method, such as that implemented to remove As (III), Cd (II), Cr (VI), Pb (II), Ni (II), Hg (II), and Cu (II), may prove to be an effective way to remove other toxic metal ions from the water in the future. Several advanced technical efficiency and environmental assessment tools can be used in the future to develop dynamic systems that will be cost-effective, energy-efficient, and environmentally friendly, such as life cycle assessments and exergo-environmental and exergoeconomic analyses of large-scale water treatment plants. Additionally, it may also be possible to examine how heavy metals are removed from the body using molecular mechanisms and comparative transcriptomic analyses in future research plans [175,176].

## 7. Conclusions

A wide range of industrial processes use heavy metal ions, including tanning and chrome plating. Among the most dangerous substances that affect human health are heavy metal ions, such as As (III), Cd (II), Cr (VI), Pb (II), Ni (II), Hg (II), and Cu (II). Several of these metal ions are released into the environment and contaminate it. In addition to human health, some aquatic and terrestrial animals are affected by heavy metal ions in water. Heavy metal toxicity is widely known to cause various types of cancer, kidney and liver damage, skin problems, and mental dullness, among others, as a result of toxic exposure to heavy metals. As part of the conventional methods for removing heavy metal ions from water, several processes are commonly used, including membrane filtration, reverse osmosis, chemical reduction, and adsorption. It is important to note that some of these methods are more costly than biological means, but they produce high amounts of secondary pollutants. Comparing eco-friendly methods with conventional methods, there is a wide gap between them in terms of advantages. Hence, biological methods can be considered the best alternative to conventional methods of heavy metals removal and play an important role in energy saving and sustainable environmental development. 

## Figures and Tables

**Table 1:** The assessment of water quality parameters and their corresponding allowable thresholds in potable water sources [32].

Parameters	Permissible Limits
pH	6–9
Temperature	25
Total solids (mg/L)	1500
Nitrate (mg/L)	50
Ammonia (mg/L)	1.5
Ni (II) (mg/L)	0.07
Zn (II) (mg/L)	0.05
Cd (II) (mg/L)	0.03 × 10^−1^
Pb (II) (mg/L)	0.01
Ti (II) (mg/L)	0.05
Cr (VI) (mg/L)	0.05
As (V and III) (mg/L)	0.01

**Table 2:** Water quality measurement methods.

Parameters	Unit	Method or Equipment Used	References
Temperature	°C	Thermometer	[39]
pH		pH meter	[39]
Nitrate	mg/L	UV spectrophotometer	[39]
Chloride	mg/L	Argentometric method (silver nitrate method) or Mohr’s method	[39]
Sodium	mg/L	Flame photometer	[40]
Total hardness	mg/L	EDTA titrimetric method	[41]
Dissolve oxygen (DO)	mg/L	Winkler method	[41]
Alkalinity	mg/L	Titrimetric method	[40,41]
Conductivity	µs/cm	Conductivity meters	[41]
Sulphate	mg/L	UV spectrophotometer	[40,41]
Phosphate	mg/L	UV spectrophotometer	[40]
Magnesium	mg/L	EDTA titrimetric method	[41]
Minerals and heavy metals	mg/L	Inductively coupled plasma optical emission spectroscopy (ICP-OES), ICP-MS, or atomic absorption spectrophotometer	[42]

**Table 3:** Heavy metal contamination and their health impacts.

Pollutants	Health Impacts	References
Pb (II)	Pb (II) is toxic to health by accumulating in the body and damages the central nervous system. Most risky relative to children and pregnant women.	[94]
As (II)	Risk of cancer and cause skin lesions. As (II) toxicity is also associated with cardiovascular diseases and diabetes.	[95]
Cd (II)	Cd (II) exposure causes reproductive, cardiovascular, pulmonary, and gastrointestinal disorders.	[96]
Cr (VI)	Cr (VI) can be responsible for acute and chronic toxicity in the living organism. It also has carcinogenic effects.	[97]
Hg (II)	It causes harmful effects on the living system, including headaches, anorexia, and rash. It also affects the digestive system, reproductive system, kidney, and respiratory system.	[98]
Ni (II)	Depending on the dosage and duration of exposure, various health issues can arise, including dermatitis, asthma, and cancer of the respiratory tract.	[99]

**Table 4:** The advantages and limitations of the heavy metal removal method.

Methods	Advantage	Limitations	References
Oxidation	A rapid process for heavy metal removal	Expensive and generates by-products	[104]
Ion exchange	Effective removal of a wide range of heavy metals	Adsorbents require regeneration or disposal	[104,105]
Chemical precipitation	An effective method for the removal of heavy metals	Production of a large amount of sludge	[104,106,107]
Adsorption	Flexibility and simplicity of method design and insensitivity to toxic metals	Regeneration required after adsorption	[104,108]
Membrane filtration	An effective method for the removal of heavy metal ions	High operation cost and concentrated sludge production	[104,109]
Photochemical	No production of sludge	Formation of by-products	[104]
Coagulation/flocculation	Economically feasible	Formation of large particles and production of sludge	[104,110]
Electrochemical coagulation	Economically feasible	A large amount of sludge production	[111]
Biological treatment	Eco-friendly, inexpensive, and effective removal of heavy metals	Biological methods have yet to be established and commercialized	[112,113]

**Table 5:** Biosorbents and their heavy metals biosorption capacity.

Biosorbent	Heavy Metal	Biosorption Capacity (mg/g)	References
*Trewia nudiflora* fruit peels powder		294.12	[132]
Banana peel	Pb (II)	0.5	[133]
Banana peel	Cd (II)	5.71	[134]
Rice husk	Cr (VI)	33.68	[135]
*Solanum melongena* leaf powder	Pb (II)	71.42	[136]
Tomato waste Apple huice residue	Pb (II)	152 108	[137]
watermelon peel waste	As (III and V)	2.42	[138]
Orange peels	As (V)	32.7	[139]
Pine cone	Pb (II) Cd (II) Cu (II) Cr (VI)	100.01 78.73 33.55 57.36	[140]

**Table 6:** Modified biosorbents and their heavy metal biosorption capacity.

Modified Biosorbent	Heavy Metals	Biosorption Capacity (mg/g)	References
$KMnO_{4}^{-}$ treated magnetic biochar	Pb (II) Cd (II)	148 79	[143]
Composite adsorbent of carrot, tomato and PET	Co (II)	312.50	[144]
Sulfuric-acid-treated orange peels	As (V)	60.09	[139]
Iron nanoparticles modified orange peels	As (V)	81.30	[145]
Ferrous-ion-doped rice husk	Cr (VI)	11.14	[146]
Microwave-assisted thiourea-modified *Sorghum* bicolor	Cu (II) Cd (II)	15.15 17.24	[147]
*Portulaca oleracea* extract fabricated Fe_3_O_4_ NPs	Cd (II) Pb (II)	177.48 108.22	[148]

**Table 7:** Microorganisms and their heavy metals removal efficiency.

Microorganism	Heavy Metals	Removal Efficiency (%)	Optimum pH	Optimum Temperature	Initial Metal Concentration (mg/L)	References
*Pseudomonas azotoformans* strain JAW1	Cd (II)	44.67	6	30	25	[157]
*Bacillus* sp. SW2 *Bacillus* sp. SW4	As (V) As (III) As (V) As (III)	53.29 51.45 51.99 50.37	-	-	100	[158]
*Bacillus* sp. Strain Q3	Pb (II)	76.4	6.2	34.3	127.4	[159]
*Paracoccus* sp. *strain NC-A* *Alcaligenes faecalis* strain NC-B *Stenotrophomonas* sp. strain NC-C	As (V) As (V) As (III)	84.50 93.00 79.60	7 7 7	35 35 35	- - -	[160]
*Bacillus cereus* S13 *Bacillus cereus* S25	Pb (II) Co (II) Cr (VI)	98.00 93.70 93.90	- - -	- - -	10 10 10	[161]

## Data Availability

Data are mentioned in this manuscript.
